# Testing patient targeted therapies in patients with temporomandibular joint disorder with the arthrokinetic reflex: individual patient research

**DOI:** 10.1186/s12967-016-0991-9

**Published:** 2016-08-02

**Authors:** Garabed G. Demerjian, Andre Barkhordarian, Francesco Chiappelli

**Affiliations:** 1 Oral Biology & Medicine, Center for the Health Sciences UCLA, Los Angeles, USA; 2 Center for TMJ and Sleep Therapy, Glendora, CA USA; 3 Evidence-Based Decisions Practice-Based Research Network (EBD-PBRN.org), Los Angeles, USA

**Keywords:** Individual patient research (IPR), Patient-targeted therapy, Temporomandibular joint disorder, Comparative effectiveness research (CER), Arthrokinetic reflex, Adaptive cluster randomized stepped wedge blinded controlled trial

## Abstract

Traditional research in the health sciences has involved control and experimental groups of patients, and descriptive and inferential statistical analyses performed on the measurements obtained from the samples in each group. As the novel model of translational healthcare, which integrates translational research and translational effectiveness, becomes increasingly established in modern contemporary medicine, healthcare continues to evolve into a model of care that is evidence-based, effectiveness-focused and patient-centered. Patient-centered care and patient-targeted therapies require the timely and critical development and validation of a new research paradigm, individual patient research (IPR), as opposed to the customary group research approach. Here, we propose a model of individual patient research to define and characterize the effectiveness of a novel therapeutic intervention for temporomandibular joint disorder. The intervention must be tailor-made for each individual patient, and the data from each patient must be analyzed individually. We propose that this endeavor is best achieved by means of an adaptive cluster randomized stepped wedge blinded controlled trial, because it permit individual patient outcomes research and analysis, ensures equipoise, and maintains adequate power. The patient targeted therapies section of the Journal of Translational Medicine must endeavor to facilitate the dissemination of studies that focus broadly on translational research for the ultimate benefit of individual patients.

## Individual patient data (IPD) in translational science

Patient-centered therapies are at the heart of translational science in healthcare, which has gained considerable momentum from the Affordable Care Act-2010. In his recent paper [1] in the Journal of the American Medical Association, President Obama wrote that “*The Affordable Care Act is the most important health care legislation enacted in the United States since the creation of Medicare and Medicaid in 1965. The law implemented comprehensive reforms designed to improve the accessibility, affordability, and quality of health care*.” That is certainly true. It is also a fact that the Affordable Care Act leaves many important questions and issues unanswered and unresolved.

Translational research and translational effectiveness unquestionably signify together a positive future for patients and stakeholders across the health sciences. Translational research utilizes laboratory protocol of basic biology and physiology on specific biopsies to better define and characterize the pathological mechanisms that underlie the patient’s condition. Translational effectiveness utilizes the research synthesis, comparative effectiveness research and meta-analysis protocols and systematic review reports to craft and establish the consensus of the best evidence base (BEB) for electing the most appropriate mode of therapeutic intervention to treat the patient’s condition [2–8].

Research synthesis, the chosen design for garnering BEB, is reported in the form of the systematic review. It is a systematic process of locating, and critically evaluating the body of research that addresses a particular clinical issue. Systematic reviews yield the highest level and quality of evidence, and the consensus of BEB for efficacy (i.e., whether the treatment under study works under the controlled conditions of randomized controlled trials, RCTs), or effectiveness (i.e., benefits, costs, risks of the intervention real life situations). BEB can be expressed qualitatively, quantitatively, and statistically. Data extraction summarizes the salient evidence, sample size, nature of the individual studies, benefits and type and frequency of adverse events [2–10]. What remains under-studied is how best can comparative effectiveness research (CER) develop and validate a new research paradigm, referred to as “individual patient research (IPR)”, as opposed to the customary group research approach.

Case in point, translational science for healthcare consists of a continuum simply described as translational research (T1), that is the transfer of knowledge from patient-centered basic research to the patient-targeted therapies. Translational effectiveness (T2), that identifies BEB from peer-reviewed clinical research, including clinical trials and observational studies, and disseminates BEB to practice settings and communities of stakeholders for the specific purpose of optimization of patient-centered, and patient-targeted interventions. This two-fold approach clearly demands increased emphasis on improving IPR, as well as optimizing patient-clinician communication and patient-tailored care delivery [2, 3]. A third level of translational science (T3) could be conceived to ensure quality improvement individual patient research (IPR) for community-based participatory and action research. This ultimate mode of translation will incorporate CER and patient-centered outcomes research and optimize dissemination and diffusion of innovations for the purpose of contributing to healthcare policies in primary care and community settings.

A *sine qua non* for patient-targeted therapies is to obtain patient-centered outcomes of research and evaluation. That is to say, we must strive to tailor our research endeavors to the needs and characteristics of individual patients, rather than pursuing the traditional route of biostatistics that describes and makes inferences of group data. To foster patient centered research in healthcare, and as a direct product of the Affordable Care Act-2010, a novel Federal entity, the Patient-Centered Outcomes Research Institute (PCORI), was formed along side the existing National Institutes of Health (NIH) and the Agency for Healthcare Research and Quality (AHRQ). As it’s principal mandates, PCORI sets the methodological standards and guidelines in patient-centered outcomes research (i.e., individual patient research, IPR), prioritizes patient-centered outcomes research questions, obtains and disseminates BEB consensus, and responds to specific critiques, input and suggestions from the stakeholders.

One example of the use of IPR in translational healthcare is the model of temporomandibular joint disorder (TMJD). We outline here this timely and critical paradigm for patient-targeted therapies in evidence-based medicine and evidence-based dentistry.

## Arthrokinetic model of temporomandibular joint disorders (TMD)

The arthrokinetic reflex describes the way in which a typical joint movement can reflexively cause neuromuscular activation or inhibition [9, 10]. In the case of the jaw joint, the synovial articulation between the temporal and the mandibular bones, the complex osteo-anatomy is compounded by the powerful masticatory musculature and extensive motor and sensory (trigeminal, V3) innervation [4, 11]. One unique feature of the joint is its unique articular disc composed of dense fibrous connective tissue, which attaches to the joint capsule, a dense fibrous membrane that surrounds the joint and is connected to it by strong ligaments, and which is positioned between the two bones that form the joint, thus creating two distinct spaces. The disc is in fact a fibrous extension of the capsule in between the two bones of the joint, which slides within the capsule during articulation of the joint driven by the neuro-musculature.

With age and trauma, the disc may become thin and undergo change of cartilage in the central part, changes that may lead to impaired movement of the joint. Among the most common disorder of the jaw joint is disc displacement, which can lead to synovial inflammation, local pain and myalgias of the face, head, neck and shoulders as well as migraine-type headaches. From the viewpoint of translational research, jaw joint biopsies can be monitored for biomarkers of proximal (i.e., synovial) and distal inflammation (i.e., saliva, peripheral blood) (e.g., interleukin-6), and neurotransmitters of pain (e.g., substance P) [12, 13].

Clinical research and observations have described the wide spectrum of the arthrokinetic reflex in TMJD, mediated largely by retrograde transport from the V3 terminal branch to the joint (auriculotemporal nerve) and the central nervous system, and which can contribute and exacerbate neuro-muscular disorders, including tourette’s syndrome, cervical dystonia, complex regional pain syndrome, gait or balance disorders, Parkinson’s disease, middle and inner ear dysfunction, impaired eye movement, sleep disturbances, pain, and related neurological symptoms [13–15]. The context of sleep is particularly important because lack of quality sleep has been associated with increased risks of several health issues including obesity, heart disease, and diabetes. Individual patient measures of sleep quality should include the patient’s quality of sleep that can be assessed with a polysomnography in an experimental sleep study, and confirmed with the two critical blood or salivary biomarkers, oxalic acid and diacylglycerol 36:3, whose levels decrease significantly following sleep deprivation and normalize upon sleep recovery [16], and functional MRI (fMRI).

**Fig. 1:**
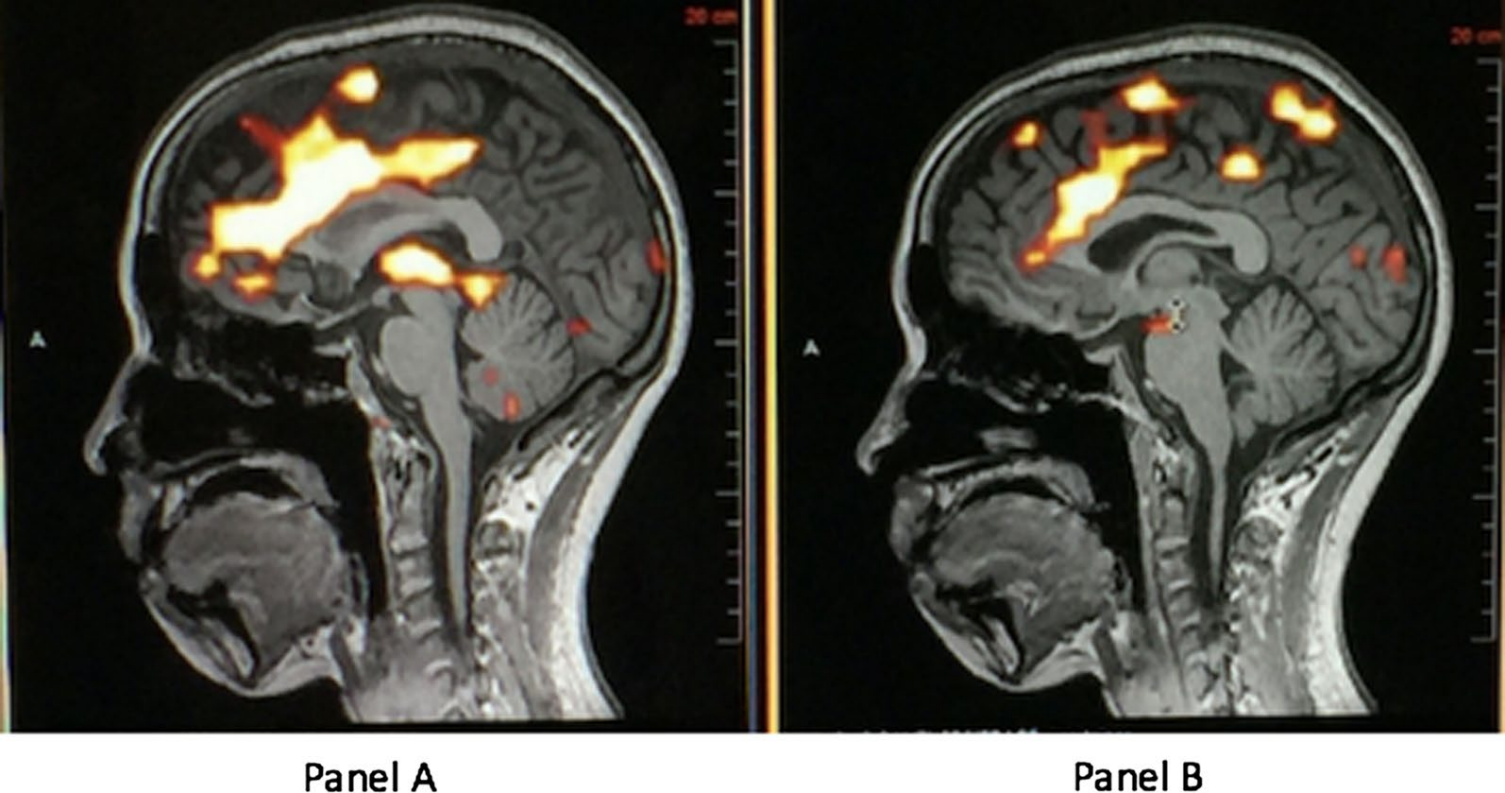
fMRI scans of one representative patient with constricted (**a**) or expanded jaw joint space (**b**). Expansion of the joint space is obtained as describe below

As proof of concept, the overarching arthrokinetic reflex in TMJD, the working hypothesis can be tested that by expanding the joint anatomical space the arthrokinetic reflex is reduced. In the context of patient-centered translational research, a broad spectrum of clinical independent patient data can be obtained from patients diagnosed clinically, by palpation as well as imaging (X-rays, CT) with mild-severe TMJD. Salivary and synovial levels of pro-inflammatory cytokines, replicate the findings reported in the literature [12], and are found to correlate with significant impairments (p < 0.05) in neuro-psychological testing (e.g., Brief Visuospatial Memory Test, Grooved Pegboard, Hopkins Verbal Learning Test, Stroop), Polysomnography and fMRI (Fig. 1), in the state of jaw joint space constriction, compared to when the joint space is expanded.

## Randomized cluster-wedge clinical trial for TMD interventions

**Fig. 2:**
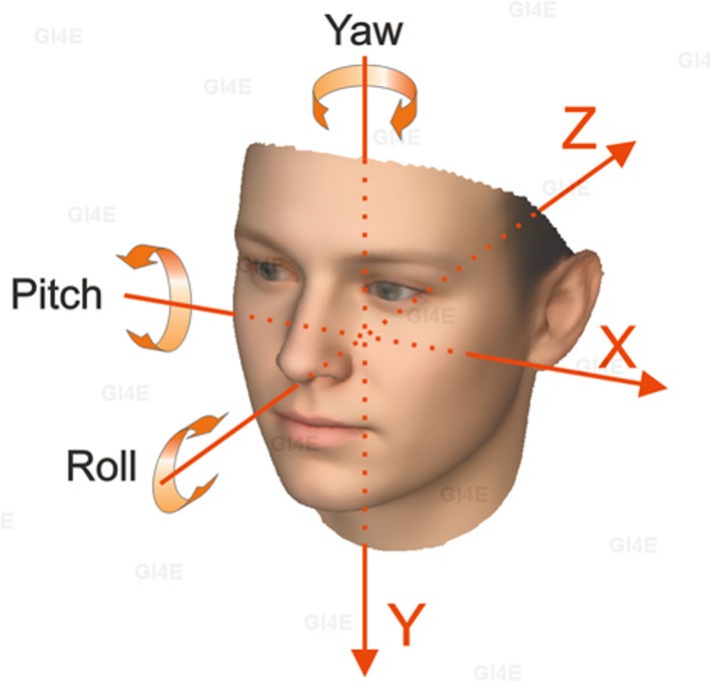
Fundamental criteria for patient-targeted therapy of TMJD

These observations strongly support the need to conduct patient-targeted therapy clinical trials to test the efficacy and the effectiveness of the intervention designed to expand jaw joint space as a potential treatment for TMJD, and more broadly for the plethora of pathologies related to the jaw joint arthrokinetic reflex. It is important to note that the intervention must be tailor-made for each patient along five principal criteria listed below and shown in Fig. 2:

- Height
- Pitch
- Yaw
- Roll
- Anterior/posterior

We have proposed elsewhere [17, 18] the potential advantages of an adaptive cluster randomized stepped wedge blinded controlled trial, that would involve sequential roll-out of the intervention in a crossover paradigm. This would allow the different clusters (i.e., ambulatory clinics of a practice-based research network) to cross over and switch treatments at different time points. The first time point would yield baseline measurements for each patient individually, as none of the clusters would be receiving the experimental intervention. Within each cluster, patients will be randomized, thus yielding a cluster randomized stepped wedge blinded controlled trial. In a design such as this, all patients eventually receive the intervention, ensuring adequate power, equipoise as well as benefit- and cost-effectiveness.

Individual patient outcome data could include salivary and synovial pro-inflammatory cytokines and pain peptides, as reported [12]. Related IPR measures could also include altered fMRI scans, and neuro-psychological testing, as our preliminary observations suggest.

Repeated individual patient outcome measures should be collected in the same manner as baseline every 3 months for 9–12 months, depending on individual patient’s clinical progress. At subsequent time points, clusters will switch over, following random ordering, and measurements would be obtained from each patient and analyzed as deltas (∆: difference from baseline). That is to say, the data of the proposed study would be—for each individual patient—the set of deltas for each variable repeated measures. Analysis of these data would follow the routine repeated measure ANOVA process, with Newman-Keuls post hoc comparisons and Bonferroni corrections for comparative purposes, and hierarchical multiple regression for predictive purposes integrating other individual patient characteristics. If the data were expressed as ratio change from baseline, then individual patient data meta-analysis could be projected with an approach similar to the present use of odds ratios in customary meta-analysis [2, 3, 6–8].

## Implications for patient-targeted therapies

This section on patient-targeted therapies of the Journal of Translational Medicine must redouble its focus on translational research (i.e., T1) investigations, on studies that pertain to translational effectiveness (i.e., T2; comparative effectiveness research), or on research that seeks to better define and characterize the T1–T2 transaction (i.e., T3). Here, we proposed a model by discussing the use of an adaptive cluster randomized stepped wedge blinded controlled trial to test a patient-targeted intervention designed to ameliorate symptomatology and pathology derived from the arthrokinetic reflex of temporomandibular joint disorder.

It is timely and critical that patient-targeted therapies be examined and tested through the critical lens of translational research, and of translational effectiveness. It is just as timely and critical that the best available evidence that derives from such translational science studies be efficiently disseminated to clinicians for the ultimate benefit of individual patient and of all stakeholders.
